# Trend analysis of hospital admissions attributable to tobacco smoking, Northern Territory Aboriginal and non-Aboriginal populations, 1998 to 2009

**DOI:** 10.1186/1471-2458-12-545

**Published:** 2012-07-24

**Authors:** Sabine LM Pircher, Shu Qin Li, Steven L Guthridge

**Affiliations:** 1 Northern Territory Department of Health, Health Gains Planning Branch, 1st Floor Darwin Plaza, Smith Street, Darwin, NT, 0810, Australia

**Keywords:** Tobacco, Smoking, Attributable, Hospital admission, Condition, Aboriginal, Trend

## Abstract

**Background:**

Tobacco smoking is a well-recognised risk factor for many diseases [1]. This study assesses the extent of smoking-attributable hospitalisation in the Northern Territory (NT) Aboriginal and non-Aboriginal populations, and examines smoking-attributable hospitalisation trends for the years 1998/99 to 2008/09.

**Methods:**

Hospital discharge data were used for the analysis. The proportion of conditions attributable to tobacco smoking was calculated using the aetiological fraction method. Age-adjusted smoking-attributable hospitalisation rates were calculated to describe the impact of tobacco smoking on the health of Territorians. A negative binominal regression model was applied to examine trends in smoking-attributable hospitalisations.

**Results:**

Aboriginal Territorians were found to have higher rates of smoking-attributable hospitalisation, with Aboriginal males more than three times and Aboriginal females more than four times more likely to be hospitalised for smoking-attributable conditions than their non-Aboriginal counterparts. The age-adjusted hospitalisation rate for Aboriginal males increased by 31% and for Aboriginal females by 18% during the study period. There were more modest increases for NT non-Aboriginal males and females (5% and 17% respectively). The increase among Aboriginal males occurred up until 2005/06 followed by moderation in the trend. There were small reductions in smoking-attributable hospitalisation rates among all populations in younger age groups (less than 25 years).

**Conclusions:**

Aboriginal Territorians experience much higher smoking-attributable hospitalisation rates than non-Aboriginal Territorians. The scale of the smoking burden and suggestion of recent moderation among Aboriginal men reinforce the importance of tobacco control interventions that are designed to meet the needs of the NT’s diverse population groups. Preventing smoking and increasing smoking cessation rates remain priorities for public health interventions in the NT.

## Background

Tobacco smoking is a well-recognised risk factor for respiratory and cardiovascular diseases and many cancers [1]. It contributes to premature death and economic loss to society, and adds a substantial burden to the Australian health-care system. While national figures on tobacco smoking prevalence show that daily smoking rates for smokers aged over 14 years have fallen from 20% in 2001 to 17% in 2007 [1], the Northern Territory (NT) has lagged behind the national trend with a smoking rate in 2007/08 of 50% for Aboriginal and 28% for non-Aboriginal populations, both higher than the national average [2]. Consistent with the high smoking prevalence, both Aboriginal and non-Aboriginal Territorians also suffer greater smoking associated morbidity [3].

To assist health promotion efforts aimed at reducing smoking prevalence in the NT, and to help reduce the excess burden attributable to tobacco smoking, up-to-date information on the effects of smoking on morbidity is needed. The aim of this study is to estimate the extent and time trend of smoking-attributable hospitalisations in NT Aboriginal and non-Aboriginal populations for the period 1998/99 to 2008/09.

## Methods

### Data sources

Inpatient de-identified unit record level discharge data for NT public hospitals were obtained from the NT Department of Health for the period from 1 July 1998 to 30 June 2009 for persons who were residents of the NT. Data were coded using the International Classification of Diseases and Related Health Problems, 10th revision (ICD-10-AM) [4]. The information presented in this study is based on the principal diagnosis only, except for fire injuries, where any diagnosis was used in the analysis. Renal dialysis patients were excluded to ensure comparability with national studies [5-7].

NT population estimates were based on 2006 Census data from the Australian Bureau of Statistics (ABS) [8].

### Method

The estimation of attributable risk of tobacco smoking in causing lung cancer was first introduced by Morton Levin. in 1953 and it formed a foundation of the aetiological fraction method [9]. English and colleagues further expended this works and included a wide range of conditions in 1995 [10]. It is a well-recognised method for quantifying morbidity due to a specified risk factor. To calculate the proportion of smoking-attributable hospitalisations, this method requires data on the prevalence of smoking and the relative risk of smokers developing a certain disease or condition [10]. Potential conditions that can be attributed to tobacco smoking have been identified in large-scale epidemiological studies [5,6,10]. These include chronic conditions and acute consequences such as trauma resulting from smoking-related accidents. The conditions included for this study are provided in Additional file 1. For the current study, passive smoking was adjusted to include children under 13 years of age [11].

Smoking-attributable conditions included in this study are partially based on the Australian Burden of Disease and Injury (ABOD) report [6]. In the ABOD report, some smoking-attributable conditions were grouped into a broader category. For the purposes of our study, these diseases were more clearly identified from earlier studies of English et al. and Ridolfo and Stevenson [10,11]. Some studies suggest that smoking provides a protective effect for ulcerative colitis, Parkinson’s disease and endometrial cancer [5]. These small benefits have been included in this study.

The relative risks for smoking-attributable conditions used in this study are largely based on English et al [10]. with subsequent updated estimates from Ridolfo and Stevenson [12] and unpublished data from the ABOD [6].

For conditions in which tobacco smoking is a contributory cause, estimates of the relative risk are combined with smoking prevalence data to derive the aetiological fraction, according to the formula: [8]

(1) $$\text{AF}=\frac{pe(RR-1)}{pe(RR-1)+1}\;$$

where

AF = aetiological fraction

p_e_ = proportion of smokers in a population

RR = relative risk of developing a disease or condition [9]

For a small number of conditions (fire injury, tobacco abuse) directly derived aetiological fractions were used.

Data on smoking prevalence by age and sex for Aboriginal Territorians were obtained from the Australian Bureau of Statistics, 2004/05 National Aboriginal and Torres Strait Islanders Health Survey [13], and for non-Aboriginal Territorians from the Australian Institute of Health and Welfare, National Drug Strategy Household Survey 2004 [14]. The availability and transparency of survey estimates of smoking prevalence was chosen for this study rather than synthetic methods proposed by Peto et al [15].

### Statistical analysis

Stata software (Version 11 – StataCorp LP, College Station, TX 77845 USA) was used for all statistical analyses. Annual smoking-attributable hospitalisation rates by sex and Indigenous status were calculated for the years 1998/99 to 2008/09 and age-adjusted using the 2001 Australian estimated resident population.

The annual change in smoking-attributable hospitalisation rates by sex and Indigenous status were estimated by means of negative binomial regression models, in preference to Poisson regression, to adjust for data overdispersion. Age-specific hospitalisation rates and time trends were further calculated to determine variations within these rates between different age-groups and different periods.

### Ethical approval

The study was approved by the Human Research Ethics Committee of the NT Department of Health and Menzies School of Health Research (HREC-2010-1335).

## Results

The number and proportion of NT smoking-attributable hospitalisations during the years 1998/99 and 2008/09 are presented in Table 1. There were 18,612 smoking-attributable hospitalisations among NT residents during the study period, accounting for nearly 4% of the 487,099 total NT public hospitalisations. There were 10,929 smoking-attributable hospitalisations among NT Aboriginal people, or 5% of the total Aboriginal hospitalisations (234,137) during the study period. Among non-Aboriginal Territorians, smoking accounted for 7,683 (3%) of 252,962 hospitalisations. Overall, more smoking-attributable hospitalisations were experienced by males (57%). Although Aboriginal people are 29% of the total NT population [8], they experienced 59% of the total NT smoking-attributable hospitalisations.

**Table 1:** Number and proportion of smoking-attributable hospitalisations, by sex and Indigenous status, NT 1998/99 to 2008/09

		**Total no. of hospitalisations**	**No. of smoking- attributable hospitalisations**	**Percent**
**Aboriginal**				
	Male	99,742	5,675	5.7
	Female	134,395	5,254	3.9
**Non-Aboriginal**				
	Male	122,926	4,941	4.0
	Female	130,036	2,742	2.1
**All NT**				
	Male	222,668	10,616	4.8
	Female	264,431	7,996	3.0

Across all years, the age-adjusted smoking-attributable hospitalisation rate in the NT Aboriginal population was 285 per 10,000 population, three times higher than the rate for non-Aboriginal people (82 per 10,000). The rate among NT Aboriginal males was 330 per 10,000 and among NT Aboriginal females, 252 per 10,000. Among NT non-Aboriginal males the age-adjusted rate was 97 per 10,000 and 62 per 10,000 among non-Aboriginal females.

Comparison of smoking attributable hospitalisation rates was made between Territorians aged under 25 years and Territorians aged 25 years and above (Table 2). The younger Aboriginal population had much higher smoking-attributable hospitalisation rates than the younger non-Aboriginal population, and in each group the males and female rates were similar. By contrast, for those aged 25 and over, males in both populations had much higher rates of smoking-attributable hospitalisations than the corresponding females. Again the rates for the Aboriginal population were much higher than the corresponding non-Aboriginal population.

**Table 2:** Age-adjusted smoking-attributable hospitalisation rates per 10,000, by sex, Indigenous status and age, NT 1998/99 to 2008/09

**Smoking-attributable hospitalisations per 10,000 population**			
		**aged <25**	**aged >=25**
**Aboriginal**			
	Male	45.2	285.3
	Female	45.5	206.4
**Non-Aboriginal**			
	Male	13.8	82.9
	Female	12.6	49.5

**Figure 1:**
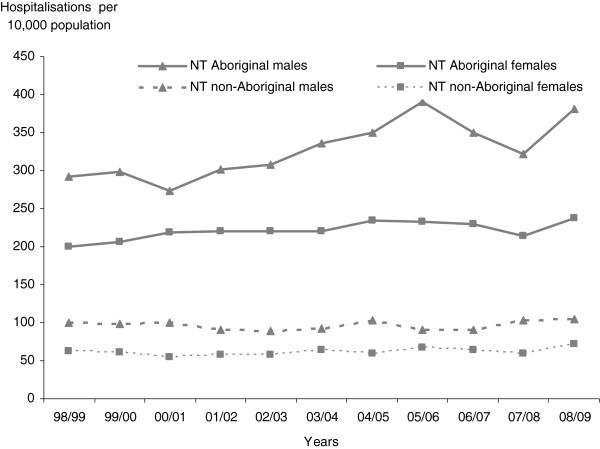
Age-adjusted smoking-attributable hospitalisation rates per 10,000, by sex and Indigenous status, NT 1998/99 to 2008/09.

Figure 1 illustrates annual age-adjusted smoking-attributable hospitalisation rates for the eleven years from 1998/99 to 2008/09. The age-adjusted smoking-attributable hospitalisation rates for Aboriginal males increased by 31% over the study period, with all of this increase occurring before 2005/06, while more recent annual rates appear to have moderated. Over the study period, the rates increased by 18% for NT Aboriginal females, and by 5% and 17% for NT non-Aboriginal males and females, respectively.

The average annual increase in the smoking-attributable hospitalisation rate for Aboriginal males was 1.2% (95%CI: -5.7, 8.8) for the years from 1998/99 to 2008/09, compared to an increase of 0.8% (95%CI:-3.7, 5.5) among Aboriginal females. For non-Aboriginal males we observed a decrease in annual average smoking-attributable hospitalisation rates of 0.7% (95%CI:-7.1, 6.0). No meaningful trend was observed for non-Aboriginal females (0.0008%, 95%CI:-4.5, 4.7).

For Territorians aged under 25 years, there was a decrease in annual average smoking-attributable hospitalisation rates among both Aboriginal and non-Aboriginal people. For young Aboriginal people this decline was mainly driven by a decrease in lower respiratory disease. For non-Aboriginal Territorians aged under 25 years, the conditions behind the decline were antepartum haemorrhage, followed by asthma and otitis media. Table 3 shows that the smallest decrease for the younger Territorians can be observed among Aboriginal females (0.4%). Among Territorians aged 25 years and over, there was an increase in annual average smoking-attributable hospitalisation rates among both Aboriginal and non-Aboriginal Territorians, with the largest increase among Aboriginal males (3.4%). There were no statistically significant trends for any of the population groups in this study (P >0.05).

**Table 3:** Annual changes in smoking-attributable hospitalisation rates, by sex, Indigenous status and age, NT 1998/99 to 2008/09

**Smoking-attributable hospitalisations**					
		**aged <25**	**95%CI**	**aged >=25**	**95%CI**
**Aboriginal**					
	Male	−1.1	(−16.7, 17.6)	3.4	(−4.9, 12.4)
	Female	−0.4	(−9.3, 9.4)	1.4	(−4.5, 7.7)
**Non-Aboriginal**					
	Male	−1.8	(−17.0, 16.2)	0.3	(−7.5, 8.8)
	Female	−2.5	(−11.1, 7.0)	2.2	(−3.7, 8.5)

The most common cause of smoking-attributable hospitalisations for Aboriginal Territorians was chronic obstructive pulmonary disease (COPD, 25%) followed by Ischaemic heart disease (IHD, 14.5%). For non-Aboriginal Territorians COPD (24.3%), IHD (16.2%) and low birth weight (8.3%) were the most common causes of smoking-attributable hospitalisations (Table 4).

**Table 4:** Number of smoking-attributable hospitalisations for the ten most common conditions, by Indigenous status, NT 1998/99 to 2008/09

**Aboriginal**		**Non-Aboriginal**	
**Conditions**	**Smoking- attributable hospitalisations**	**Conditions**	**Smoking- attributable hospitalisations**
COPD	2,727	COPD	1,869
Ischaemic heart disease	1,580	Ischaemic heart disease	1,246
Lower respiratory illness	1,412	LBW	639
LBW	1,348	Lung cancer	543
Heart failure	951	Cardiac dysrhythmias	542
Stroke	489	Stroke	419
Fire injuries	356	Asthma, aged <15 years	297
Cardiac dysrhythmias	348	Fire injuries	260
Premature rupture of membranes	337	Premature rupture of membranes	256
Lung cancer	253	Heart failure	217

Of the ten most common conditions the greatest increase in hospitalisations during the study period was for lung cancer, which increased more than three times among Aboriginal Territorians and by almost three times among non-Aboriginal Territorians. Among non-Aboriginal people smoking-attributable hospitalisations for asthma almost halved and also decreased for low birth weight.

## Discussion

Despite vigorous anti-smoking campaigns initiated by both government and non-government agencies, Territorians experience much higher smoking-attributable hospitalisation rates than other Australians [3,16]. This result is consistent with the higher rate of smoking among the NT Aboriginal and non-Aboriginal population [17]. Smoking-attributable hospitalisation studies in the general Australian population have not used consistent methods and comparisons between time periods have not been reported. In the NT, a study by Measey et al. reported that between 1993 and 1995 NT Aboriginal males were more than twice as likely and NT Aboriginal females more than four times as likely to be hospitalised for a smoking related condition compared with their non-Aboriginal counterparts [3]. Our results show that for the period from 1998/99 and 2008/09 these differences have increased among males, with NT Aboriginal males now more than three times more likely to experience smoking-attributable hospitalisations, while the ratio for NT Aboriginal women remains four times that of their non-Aboriginal counterparts. We may however have reached a peak, at least among Aboriginal men with early signs of moderation of the trend of increasing annual smoking-attributable hospitalisation rates. It is too early to demonstrate a firm change in trajectory but a moderation in hospitalisation rates is consistent with the recent reports that smoking prevalence may be falling among Aboriginal men [17,18].

Of specific concern from this study is that despite focus in the NT on improvements in antenatal care there was only a marginal decrease of smoking-attributable hospitalisation rates among Aboriginal females aged under 25 years between 1998/99 and 2008/09. This is of particular concern as these young women may be pregnant or raising children. Smoking in pregnancy is known to be a major risk factor for preterm delivery, complications in childbirth, fetal growth restriction, stillbirth, low birth weight and infant mortality [19].

The high smoking prevalence in the NT, particularly among the Aboriginal population poses an ongoing challenge, not only for health and health services, but also economically. There is clear evidence for the benefit of health promotion interventions targeted at prevention, legislation and smoking cessation, preventing smoking and increasing cessation rates and these need to remain priorities for public health professionals. However, the differential impact of such interventions for Aboriginal people remains poorly understood [20]. Higher rates of smoking-attributable hospitalisations among Aboriginal Territorians highlight the importance of tobacco control interventions that are specifically designed to meet the needs of NT’s diverse population.

Because factors that contribute to smoking prevalence occur at both, the community level, as well as at an individual level [21,22], a community-based approach that targets social issues, economics and policies for prevention and cessation is likely to be effective over time. In addition to community strategies, interventions need to address high risk groups such as young Aboriginal women to prevent them from taking up smoking. The NT Tobacco Action Plan 2010–2013 is a framework for reducing tobacco-related harm. It includes interventions that address policy and legislation, health care and community interventions [23]. Recent amendments to the NT Tobacco Control Act have resulted in all public outdoor eating and drinking areas in the NT becoming legally smoke-free [23]. In addition, owners of outdoor public areas are now able to declare the area smoke free, licensing arrangements have been reinforced and the display of all tobacco products at the point of sale is prohibited [23].

### Limitations

There are a number of recognised limitations to this study. Firstly trends in hospitalisation rates should be interpreted cautiously, as they are influenced by changes in admission practices and disease management. Secondly, data on admissions to the single NT private hospital were not available at the time of this study. While most hospital services to Aboriginal Territorians are provided by public hospitals [24], the absence of private hospital data may have resulted in underestimation of smoking-attributable hospitalisations among the NT non-Aboriginal population. Thirdly, the relative risks applied in this study are based on those from previous studies in the general population [5,6,9]. There are no specific relative risks available for Aboriginal populations and we therefore have assumed that the excess risk is the same across all Territorians. Even though the effects of smoking-attributable diseases are mainly a result of biological mechanisms, other factors such as the living environment also influence the strength of the relationship between smoking and a certain condition [3]. The real risks may vary between different populations.

There are also limitations to the measures of prevalence used in this study. Apart from differences in survey methods, factors such as age at which smoking began, duration of smoking, the number of cigarettes smoked per day, nicotine content or filter type, and different behaviours influence the results of surveys in smoking prevalence [25]. Due to the time lag between smoking prevalence and the onset of many diseases, caution is further required when interpreting the results. The time lag varies widely between conditions, and in most cases is cumulative, and partly reversible. Using a single smoking prevalence estimate in the study may have resulted in an underestimate of the results given that smoking prevalence among Aboriginal Territorians may have increased over the study period [18]. Differences in prevalence alone are however not entirely responsible for the differences between Aboriginal and non-Aboriginal smoking-attributable hospitalisation rates. When applying the aetiological fraction method to Aboriginal Territorians, the results need to be considered in context with the compounding effect of the extremely high morbidity among this population group. It is well recognised that a combination of low socioeconomic status, poor nutrition, and rural and remote dwelling and many other risk factors contribute to the development of chronic diseases for which smoking may be a major cause.

## Conclusions

Aboriginal Territorians experience much higher smoking-attributable hospitalisation rates than non-Aboriginal Territorians. The scale of the smoking burden and suggestion of recent moderation among Aboriginal men reinforce the importance of tobacco control interventions that are designed to meet the needs of the NT’s diverse population groups. Preventing smoking and increasing smoking cessation rates remain priorities for public health interventions in the NT.

## Competing interests

The authors declare that they have no competing interests.

## Author’s contributions

SLMPhas made substantial contributions to the design of the study, analysis and interpretation of the data, drafting the manuscript. SQL has made substantial contributions to the design of the study, supervised the data analysis and interpretation of the results, drafting the manuscript. SLG has made substantial contributions to the design of the study, interpretation of the result and editing of the manuscript. All authors read and approved the final manuscript.

## Pre-publication history

The pre-publication history for this paper can be accessed here:

http://www.biomedcentral.com/1471-2458/12/545/prepub

## Supplementary Material

Additional file 1 Appendix A. Causes of death and principal diagnoses identified as tobacco-related conditions. Click here for file
